# Comparison of The Effects of Vitrification on Gene Expression of Mature Mouse Oocytes Using Cryotop and Open Pulled Straw

**DOI:** 10.22074/ijfs.2018.5112

**Published:** 2018-01-05

**Authors:** Fardin Amidi, Zahra Khodabandeh, Mohamad Hossain Nori Mogahi

**Affiliations:** 1 Department of Anatomical Sciences, Tehran University of Medical Sciences, Tehran, Iran; 2 Stem Cells Technology Research Center, Shiraz University of Medical Sciences, Shiraz, Iran; 3 Shiraz Institute for Stem Cells and Regenerative Medicine, Shiraz University of Medical Sciences, Shiraz, Iran

**Keywords:** Cryotop, Gene Expression, Oocyte, Vitrification

## Abstract

**Background:**

Oocyte cryopreservation is an essential part of the assisted reproductive technology (ART), which was recently introduced into clinical practice. This study aimed to evaluate the effects of two vitrification systems-Cryotop and Open Pulled Straw (OPS)-on mature oocytes gene expressions.

**Materials and Methods:**

In this experimental study, the survival rate of metaphase II (MII) mouse oocytes were assessed after cryopreservation by vitrification via i. OPS or ii. Cryotop. Then we compared the fertilization rate of oocytes produced via these two methods. In the second experiment, we determined the effects of the two vitrification methods on the expression of *Hspa1a, mn-Sod*, and *ß-actin* genes in vitrified-warmed oocytes. Denuded MII oocytes were vitrified in two concentrations of vitrification solution (VS1 and VS2) by Cryotop and straw. We then compared the results using the two vitrification methods with fresh control oocytes.

**Results:**

*mn-Sod* expression increased in the vitrified-warmed group both in OPS and Cryotop compared with the con- trols. We only detected *Hspa1a* in VS1 and control groups using Cryotop. The survival rate of the oocytes was 91.2% (VS1) and 89.2% (VS2) in the Cryotop groups (P=0.902) and 85.5% (VS1) and 83.6% (VS2) in the OPS groups (P=0.905). There were no significant differences between the Cryotop and the OPS groups (P=0.927). The survival rate in the Cryotop or the OPS groups was, nevertheless, significantly lower than the control group (P<0.001). The fertilization rates of the oocytes were 39% (VS1) and 34% (VS2) in the Cryotop groups (P=0.902) and 29 %( VS1) and 19.7% (VS2) in the OPS groups (P=0.413). The fertilization rates were achieved without significant differences among the Cryotop and OPS groups (P=0.755).

**Conclusion:**

Our results indicated that Cryotop vitrification increases both cooling and warming rates, but both Cryo- top and OPS techniques have the same effect on the mouse oocytes after vitrification.

## Introduction

Oocyte cryopreservation is an essential part of the assisted reproductive technology (ART), which has been recently introduced into clinical practice. Additionally, this method is useful for the preservation of female genetic resources through oocyte banking (1, 2). The cryopreservation of the mammalian oocyte has proven to be more difficult than other cell types because of its sensitivity towards ice crystal formation and the sensitivity of meiotic spindle to changing temperature during the process of freezing and thawing (3). The freezing and thawing cause meiotic spindle destruction; therefore, it is essential to incubate the oocytes for 3-5 hours post-warming. Then, the meiotic spindle can regenerate (4, 5). Vitrification is a practical method that produces a glass-like solidification of the cells by rapid cooling and high concentrations of cryoprotective agents (CPAs). Consequently, this method can decrease the formation of ice-crystals and cell injury (6, 7).

Different types of cryoprotectants are used for vitrification protocols, including ethylene glycol (EG), dimethyl sulfoxide (DMSO), and 1, 2-propanediol (PrOH). EG is a common CPA that is used for oocyte vitrification. DMSO and PrOH are used regularly as permeating CPAs to cryopreserve oocytes and embryos to prevent the intra-cellular ice crystal formation. The combinations of CPAs can decrease the concentration of each CPA, as well as diminishing the toxic effects of CPA on the oocytes (8, 9). Non-penetrating CPAs, such as sucrose, are often used in combination with other permeating CPAs to prevent ice crystal formation and decease the CPA toxicity (10).

There are many vitrification devices that increase the cooling rate, such as cryoloop, solid surface (11), Cryotop (12), and open pulled straw (OPS) (13). The Cryotop consist of a hard plastic and a fine thin filmstrip (14). The minimum amount of vitrification solution (~0.1 µl) remaining in Cryotop is in direct contact with liquid nitrogen during cooling. As a result, ice crystal formation is prevented due to dramatically increased cooling rate (12). OPS vitrification is another popular method for human oocyte and embryo vitrification (15-17). OPS has a small effect for reducing the volume of vitrification solution to 0.5 µl and thus increasing the cooling rate (18). In recent studies, the advantages of Cryotop was compared with OPS in different species, including pig (19), human (20), and matured bovine oocytes (15). However, additional information is required to identify the effect of these two devices on mouse oocytes (21).

Other studies have reported that the structural and morphological injures occur in the vitrified-warmed oocytes. These include zona hardening, variation in selective permeability of plasma membrane, aneuploidy, and nuclear fragmentation (8, 6, 22). Vitrification may also result in changes at the molecular level in vitrified oocytes. Heat shock protein *(Hsp) a1a* and the manganese super oxide dismutase (*mn-Sod*) are two critical genes related to stress. Hsps play a protective function against heat, stress response, or both in cellular auto-regulation. The critical role of *Hspa1a*, as a defensive protein resulting from external stress, has been proven. It is confirmed that knock-out Hsp70.1 mice have higher sensitivity to osmotic stress after preconditioning them with heat (23, 24). Hut et al. (25) showed that *Hspa1a* has a protective effect on the mitotic cell cycle against heat-induced centrosome damage, preventing chromosomal division. *Mn-Sod* is an anti-oxidant enzyme that protects the oocytes and embryos against the oxidative stress damages. It was stated that adding antioxidant enzymes such as catalase or *Sod1 (Cu-Zn-Sod)* to culture media leads to an improved rate of blastocyst formation in rabbit (26), and mouse (21). Sonna et al. (27) reported that cold stress can influence the expression of genes associated with stress (stress-response genes).

In this study, the effect of vitrification protocols on the oocyte’s gene expression was investigated using mature mouse oocytes. Hence, the efficiency of the two vitrification methods (OPS vitrification to Cryotop method) was compared on fertilization percentage, morphological survival, and gene expression of *Hspa1a* and *mn-Sod* in the mouse oocytes.

## Materials and Methods

The present experimental study was conducted using mouse oocytes and sperm. The study protocol was approved by the Research Ethics Committee of Tehran University of Medical Sciences. All chemicals and media were purchased from Sigma-Aldrich Co (St.Louis, Mo, USA), unless otherwise mentioned.

### Experimental design

The fertilization rate of metaphase II (MII) mouse oocytes was assessed after cryopreserving by vitrification using: i. OPS or ii. Cryotop. In the second experiment, we determined the effects of two vitrification methods on the oocytes gene expression.

### Experiment 1

Mature oocytes were randomly selected and distributed amongst three experimental groups (OPS, Cryotops, and controls). All vitrification groups were divided into VS1 (10% v/v cryoprotectants) and VS2 (14.5 %v/v cryoprotectants) subgroups and a total of 119 and 114 were OPS-vitrified in VS1 and VS2. Also, 135 and 136 were cryotop-vitrified in VS1 and VS2; finally, 136 oocytes were used as controls. After vitrification and warming, the oocytes in all groups were fertilized and cultured *in vitro*.

### Experiment 2

The oocytes were analyzed by reverse transcription- polymerase chain reaction (RT-PCR) to evaluate changes in *Hsp70* and *mn-Sod* expression in all groups.

### Oocyte collections

Female NMRI mice aged 8 to 10 weeks were kept under 12 hours of light/dark condition. The female mice were superovulated by intraperitoneal (i.p.) injection of 10 IU pregnant mare’s serum gonadotropin (PMSG), followed by i.p. injection of 10 IU human chronic gonadotropin (hCG) 48 hours later. The mice were sacrificed by cervical dislocation 13-15 hours post-hCG administration (6). The cumulus-oocyte complex (COC) were collected from the oviduct and oocytes denudation were performed using 300 µg/ml hyaluronidase in hepes-buffered TCM199 for 30 seconds. The normal mature oocytes were selected with first polar body, intact zona pellucida, and plasma membrane.

### Preparation of vitrification and dilution solution

TCM199 supplemented with 20% fetal bovine serum (FBS) were used as a base medium. The first vitrification solution (VS1) consisted of 10% EG, 10% DMSO, and 0.5 M sucrose in the base medium (21). The second vitrification solution (VS2) was contained 14.5% EG+14.5% PrOH and 0.5 M sucrose in the base medium. The equilibration solution included (ES1) 5% EG and 5% DMSO without sucrose in the base medium and the second equilibration solution (ES2) contained 7.25% EG+7.25% PrOH without sucrose in the base medium. Warming solution (WS) contained 1 M sucrose in the base medium, and diluents’ solution (DS) contained 0.5 M (DS1) and 0.25 M (DS2) sucrose, respectively. All vitrification process steps were performed at room temperature (25°C) (13, 15).

### Occyte vitrification/warming

The COC were isolated from 32 female mice by simple random sampling. Then, the denuded MII oocytes were vitrified in two concentrations of VS1 and VS2 by Cryotop and OPS (13). Oocytes at VS1, VS2, and control groups were exposed to the first equilibration drop for 3 minutes and then the first drop was merged with adjacent ES drop. Subsequently, the oocytes were incubated in vitrification solution, VS1 and VS2, each one for less than 1 minute. Every five oocytes were quickly loaded on the top of per Cryotop (Kitzato, Ltd, Japan, Cryotop group) or loaded into OPS. Excess media were carefully removed around the oocyte in the Cryotop and then immediately submerged in liquid-nitrogen (LN2). OPS was also sealed and plunged directly into LN2. The oocytes were stored in LN2 for 7 days.

**Fig.1:**
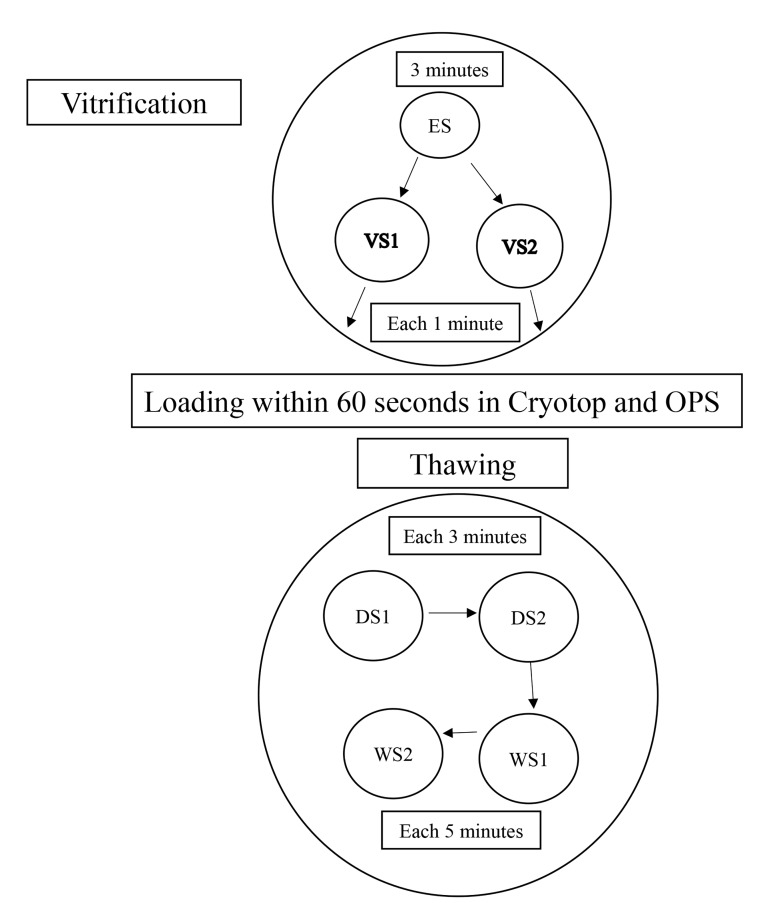
A schematic of vitrification and warming procedure.

**Fig.2:**
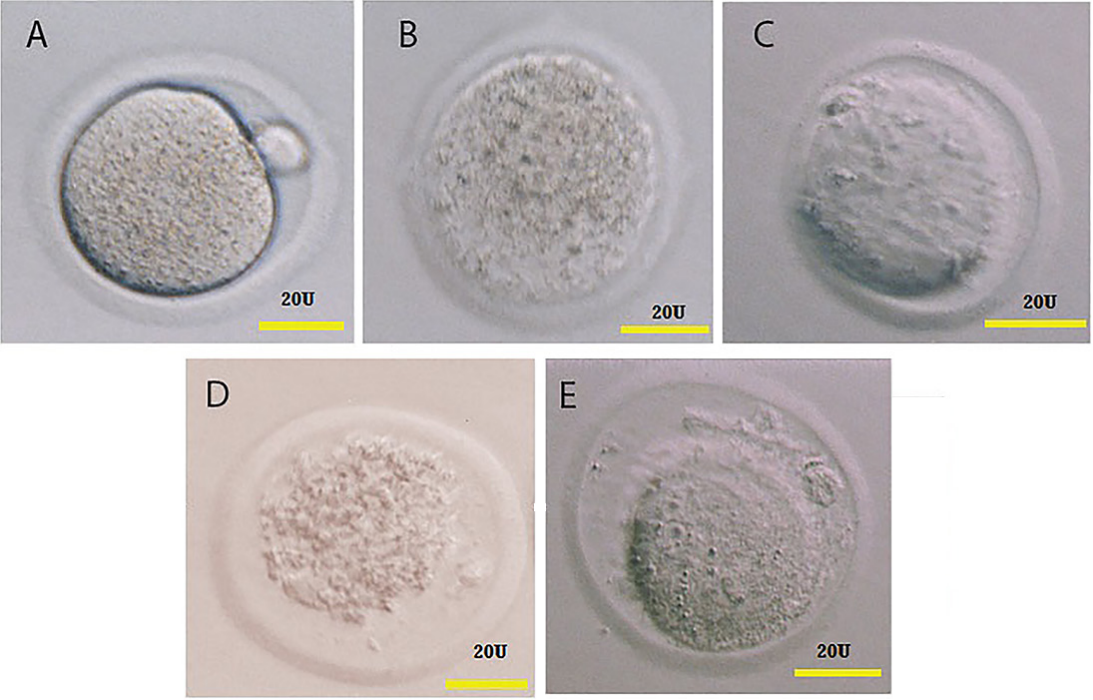
Morphology of vitrified MII oocytes after warming. Oocyte vitrified in two vitrification solution (VSI and VSII) by OPS and Cryotop. A. Control, B. VSI, Cryoptop, C. VSII, Cryotop, D. VSI, OPS, and E. VSII, OPS 20 U means 20 micron). MII; Metaphase II, VS; Vitrification solution, and OPS; Open Pulled Straw.

During warming, the Cryotop was immediately inserted into WS at 37°C for 1 minute (Cryotop group) or the straw was taken out and immersed into 37°C water for 30 seconds. The straw end was cut and its contents were transformed into a drop of 1 M sucrose (straw group). Then, the oocytes were placed onto decreasing sucrose concentrations (DS1 and DS2) to remove cryoprotectants, for 3 minutes each. Finally, the warmed oocytes were washed twice in the base medium using WS, each time for 5 minutes (Fig .1). We assessed the survival rates of vitrified-warmed oocytes on the basis of normal appearing zona pellucida and intact polar body (Fig .2). After warming, groups of 15 oocytes were stored at -80°C in Tripure isolation reagent for RNA extraction and groups of 15 oocytes were also incubated in the base medium before *in vitro* fertilization (IVF).

**Table 1:** The Primer sequences for reverse transcription-polymerase chain reaction


Gene	Gene bank accession number	Primer sequencing (5ˊ-3ˊ)	Annealing temperature (ºC)	Location	Size bp

*ß-actin*	NM_001101	F: tcatgaagatcctcaccgag	60	650-839	190
		R: ttgccaatggtgatgacctg			
*Sod2*	NM_001024466.1	F: ggaagccatcaaacgtgact	55	237-398	161
		R: ccttgcagtggatcctgatt			
*Hspa1a*	ENST00000375651	F: cgacctgaacaagagcatcaac	59	668-862	194
		R: tgaagatctgcgtctgcttggt			


### *In vitro* fertilization

The vitrified/warmed oocytes with intact zona pellucida, intact plasma membrane plus homogeneous cytoplasm were chosen and placed in 200 µl drops of IVF medium [human tubal fluid (HTF)+15 mg/ml bovine serum albumin (BSA)] layered under mineral oil (Sigma, 8410). The medium was prepared earlier to equilibrate and incubated at 37°C in 5% CO_2_ for 2 hours. A suspension of epididymal spermatozoa was prepared and the sperms were capacitated in the medium (HAM's F10+4 mg/ml BSA) at 37°C in 5% CO_2_ for 45-60 minutes. A final concentration of 2×10^6^ spermatozoa/ ml was added to IVF medium containing 15 oocytes and incubated at 37°C in 5% CO_2_ for 6 hours. Finally, the oocytes that developed into pronuclear stage were used for fertilization.

### RNA isolation and reverse transcription

Total RNA was extracted from the vitrified and non- vitrified oocytes. A number of oocytes were lysed with Tripure isolation reagent (Roche, Germany), according to the manufacturer’s instructions. The concentration and purity of the extracted RNA were determined by ND-1000 spectrophotometer (Nanodrop, USA). To synthesize cDNA, we used 300 ng/µl of total RNA and cDNA Synthesis Kit (Bioneer, South Korea) by following the manufacturer’s protocols.

### Polymerase chain reaction

RT-PCR was performed using Taq polymerase enzyme (Roche). Reactions (25 µl) contained 1 µl of each primer mix, 2 µl dNTP, 2.5 µl 10X buffer with MgCl_2_, 0.3 µl rTaq polymerase enzyme, 1 µl cDNA, and 18.2 µl DEPC water in every well. The initial denaturation step was 3 minutes at 94°C and then denaturation in each cycle was 30 seconds at 94°C. Then annealing was done for 30 seconds at 55°C for *mn-Sod*, and 59°C for *Hspa1a* and it was extended for 1 minute at 72°C. Expression of *ß-actin* housekeeping gene was used as a reference for the level of target gene expression.

PCR primers were designed using primer 3 software based on mouse DNA sequences found in the Gene Bank (NCBI) (Table 1) (28). The primers were placed into BLAST search to examine the aligned sequences for polymorphisms and avoided these regions for primers or probe design. RT-PCR products were electrophoresed on a 2% agarose gel. After stained by ethidium bromide (Cina Gene), the products were then visualized under ultraviolet. The no template control (NTC) includes all the RT-PCR reagents except that the template was considered as a negative control. A run on 2% agarose and no DNA band was also visualized (data was not shown).

### Statistical analysis

Oocyte survival and fertilization rates were analyzed by SPSS version 16 software package. All percentages of values were subjected to arcsine transformation prior to analysis. All data were expressed based on mean ± SEM. The level of statistical significance was set at P<0.05.

## Results

### Vitrification and *in vitro* fertilization

The survival of the vitrified/warmed oocytes were assessed according to their morphology in the control, the Cryotop, and the OPS groups (Fig .2). There was no difference in oocyte survival between the VS1 group and the VS2 group when using the Cryotop method. Similarly, there was also no significant difference in oocyte survival between the VS1 and the VS2 group when oocytes were vitrified by the OPS method (P=0.905). There were also no significant differences in oocyte survival between oocytes vitrified by the Cryotop and the OPS methods within the same vitrification solution group (P=0.927). The survival rate in the Cryotop or the OPS groups was, nevertheless, significantly lower than the control group (P<0.001).

The results showed a significant reduction in the fertilization rate of each group in comparison with the control (P<0.05). There is also no significant difference in oocyte fertilization between the VS1 and the VS2 group when oocytes were vitrified by Cryotop (P=0.902). There is also no significant difference in oocyte fertilization between the VS1 and VS2 the group when oocytes were vitrified by OPS method (P=0.413). The fertilization rates were achieved without significant differences among the Cryotop and the OPS groups (P=0.755, Table 2).

**Table 2:** Effects of two different vitrification solutions on the survival and the fertilization rate of the MII oocytes


	Survival Mean ± SEM			Fertilization Mean ± SEM		
Device	Control	Cryotop	OPS	Control	Cryotop	OPS

Control	100 ± 0.001			88.0 ± 2.3 (131/136)		
VS1		91.2 ± 6.7	85.5 ± 1.2		39.0 ± 5.8 (58/135)	29.2 ± 2.4 (57/119)
VS2		89.2 ± 6.1	83.6 ± 1.19		34.0 ± 5.7 (48/133)	19.7 ± 2.3 (49/114)
P value		0.004			0.001	


Tukey’s method was used for multiple comparisons. No significant differences were detected amongst the treatment groups (P<0.05). The experiments were replicated 3 times. MII; Metaphase II, VS; Vitrification solution, and OPS; Open Pulled Straw.

### Gene expression analysis

#### Cryotop groups

**Fig.3:**
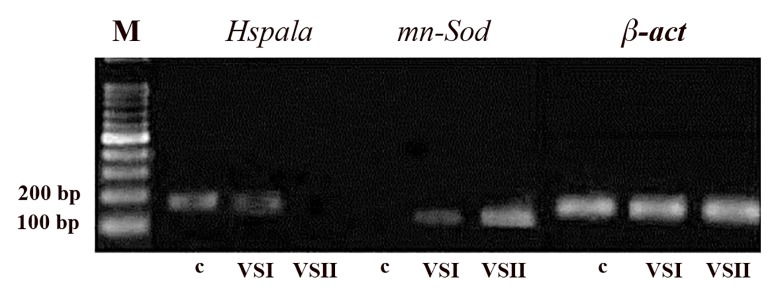
The expression of *Hspa1a* and *mn-Sod* genes was examined by reverse transcriptase- polymerase chain reaction; then, products run on 2 percent agarose gel (Cryotop groups). M; Marker, c; Control, and VS; Vitrification solution.

The expression of all genes in the vitrified-warmed oocytes in Cryotop was compared to the control (Fig .3). RT- PCR was prepared to investigate the alternation in gene expressions. The abundance of mRNA declined in the oocytes as a by-product of the vitrification procedures, but the expression of *mn-Sod* increased in the vitrified- warmed oocytes in comparison with the control group. We also detected *Hspa1a* in the control and VS1 in the Cryotop group. 

#### Open Pulled Straw groups

**Fig.4:**
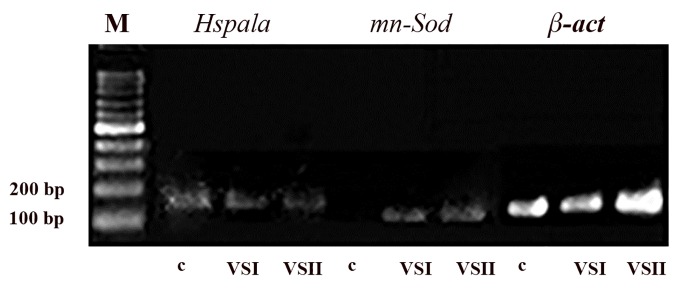
The expression of *Hspa1a* and *mn-Sod* genes was examined by reverse transcriptase- polymerase chain reaction; then, products run on 2% agarose gel [Open Pulled Straw (OPS) groups]. M; Marker, c; Control, and VS; Vitrification solution.

The expression of *Hspa1a* and *mn-Sod* was assessed in the OPS group and compared to the control group. The results presented in Figure 4 show that *Hspa1a* was expressed in the VS1, the VS2; and the control groups, but *mn-Sod* was expressed only in the VS1 and the VS2 groups.

## Discussion

In the present study, we observed that the Cryotop or the OPS changed the expression levels of a Hsp70 family (*Hspa1a*), and an antioxidant enzyme (*mn-Sod*) in the vitrified- warmed MII-oocytes. The results showed that there were no a significant differences between the quality of the Cryotop and the OPS methods in the morphology and the fertilization rates in mouse MII oocytes. Significant decreases in the fertilization rate of the vitrified-warmed oocytes compared to the control in both the VS1 and the VS2 groups were observed regardless of the vitrification methods.

Optimal cryopreservation can be achieved by limiting the two essential factors in various vitrification protocols: chilling injury and ice formation (15). To minimize the chilling injury, the vitrification procedure can use high cooling rate. This can be achieved by minimizing the volume of vitrification solution and direct contact between the sample and liquid nitrogen. Furthermore, in the vitrification protocol, high concentrations of CPAs were used to avoid ice crystal formation, but the cytotoxicity and the osmotic stress were increased. Permeating cryoprotectants were used to prevent intracellular ice crystal formation. Therefore, the use of various CPAs combinations can be efficient in reducing the concentration and the individual- specific toxicity of each CPA (29).

Vitrification process can induce stress. Hence, it is critical to choose an appropriate approach in order to minimize oxidative, osmotic, and heat stress (23). In this study, we attempted to increase the cooling rate by using a minimum volume cooling method (Cryotop) or the OPS, and then compare them with each other. It has been demonstrated that a high cooling rate reduces the toxicity of high CPAs concentrations, thus minimizing the oxidative stress and also improving the efficiency of cryopreservation (18, 30). In this study, we compared the Cryotop and the OPS vitrification, two popular minimum volume vitrification methods that provide high cooling rates, for mouse oocyte cryopreservation. The results demonstrated that the efficacy of both methods to allow mouse oocytes to undergo normal fertilization after warming.

Cryotop vitrification has been a widely used method for oocyte vitrification. Previously, we reported that using the Cryotop vitrification with a mixture of 15% EG and 15% DMSO is beneficial for vitrifying oocytes (30). Chian et al. (8) and Habibi et al. (31) also obtained a high survival rate of *in vitro* matured bovine oocytes vitrified by the Cryotop method using various combination of CPAs. In this study, oocytes vitrified by the Cryotop method resulted in a higher survival rates compared with those vitrified by OPS method. However, the differences were not significant. These results were in agreement with a previous report that compared the two vitrification methods (the Cryotop and the OPS) using calf and cow oocytes with different combinations of CPAs (15).

In addition to evaluating the effects of the vitrification methods on the oocyte viability, we also assessed the *Hsp70* and *mn-Sod* expression in the oocytes vitrified by the OPS or the Cryotop. Based on the works done on the animal models, reduced fertilization rate and low competency of the oocytes after warming may be associated with alternation in expressions of antioxidant enzymes and also hereditary factors in the oocytes (32, 33), as well as the toxicity of cryoprotectants. The development of the oocytes is dependent on the presence of specific transcripts (34).

The selected genes were involved in response to stress (*mn-Sod*, and *Hspa1a*). Changes in gene expression are considered as an integral part of cellular response to thermal stress. It is widely accepted that Hsps, whose expression is affected by heat shocks, are the best candidate. It was recently indicated that thermal stress can induce expression in a number of non-Hsps genes like *mn-Sod* (25, 29).

*Hspa1a* is a member of the inducible heat-shock family that can protect the oocytes against oxidative stress (35). In the present study, we only detected *Hspa1a* in the control and the VS1 group in the Cryotop groups, but *Hspa1a* was expressed in both the VS1 and the VS2 as well as the controls in the OPS groups. Boonkusol et al. (36) reported a similar result after vitrification with straw. The difference in gene expression observed in present study suggests that different vitrification methods may in affect the oocytes differently at the molecular level.

Oxidative stress may weaken the intracellular function and affect further development of the oocytes. Oxidative stress caused DNA instability in the mouse oocyte (37). Moreover, Bilodeau et al. (38) reported that during cryopreservation, the activity of Sod was reduced by 50% in bovine spermatozoa. Therefore, high expression of *mn-Sod* in the vitrified-warmed oocytes can be a defense mechanism against oxidative stress. In the present study, the expression of *mn-Sod* was increased in both the VS1 and the VS2 in the Cryotop and the OPS groups. We found that the survival rate and the developmental competence of the mouse MII oocytes after being vitrified both in 10% EG+10% DMSO mixture and 14.5% EG+14.5% PrOH in the Cryotop and the OPS groups showed the same effects.

## Conclusion

Our findings confirmed that the Cryotop and the OPS both can be a good candidate in mouse oocytes vitrification. It is crucial to perform further studies focusing on the expression patterns of the genes involved in early differentiation stages.
